# Sensitivity factors for diffuse optics – derivation of equations for finite changes

**DOI:** 10.1364/BOE.591980

**Published:** 2026-07-29

**Authors:** Aleh Sudakou, Stanislaw Wojtkiewicz, Roman Maniewski, Adam Liebert

**Affiliations:** Nalecz Institute of Biocybernetics and Biomedical Engineering, Polish Academy of Sciences, Warsaw, Poland

## Abstract

We present analytical equations for time-resolved signals in diffuse optics and their sensitivity factors in homogeneous media, derived from perturbation-based equations valid for infinitesimal changes and then extended to finite changes in optical properties. Time-resolved optical measurements are becoming increasingly available due to technological advancements, expanding their use in various techniques such as near-infrared spectroscopy (NIRS). These measurements acquire distributions of times of flight (DTOF) of photons, and the measurands considered in this study are attenuation (*A*), mean time of flight (*m*_1_), and variance (*V*) of the DTOF. Established methods for recovering changes in the absorption coefficient (Δ*μ*_a_) from Δ*A*, Δ*m*_1_, or Δ*V* use perturbation-based sensitivity factors, which are valid for infinitesimal changes. We derived sensitivity factor equations that relate Δ*A*, Δ*m*_1_, and Δ*V* to finite (including large) changes in absorption (Δ*μ*_a_), reduced scattering coefficient $\left(\Delta \mu_{s}^{\prime }\right)$ , and source-detector distance (Δ*r*). The derivations rely on solutions of the diffusion equation (DE) for homogeneous infinite (IM) and semi-infinite (SM) media within the diffusion approximation $\left(\mu_{s}^{\prime }\gg \mu_{a}\right)$ , without introducing additional assumptions. We also present analytical equations for the modified Beer–Lambert law (MBLL) that are valid for finite Δ*μ*_a_ in IM and SM, which can be directly applied in continuous-wave NIRS data analysis. Sensitivity factors and the MBLL require knowledge of the baseline optical properties and the differential pathlength factor (*DPF*), and we assessed how errors in these parameters affect the recovered Δ*μ*_a_. We also assessed cross-talk, in which scattering changes $\Delta \mu_{s}^{\prime }$ lead to spuriously recovered absorption changes Δ*μ*_a_, and vice versa. The proposed framework can be used to improve the accuracy of methods for estimating changes in optical properties.

## 1. Introduction

Perturbation theory is a widely used mathematical approach for deriving equations that relate changes in a measurand to changes in a parameter by linearizing their relation, enabling recovery of the parameter change from the measured signal change. When the relation is nonlinear, such equations are valid only for infinitesimal changes. When approximating a nonlinear dependency by a linear function, the deviation on the left side of the baseline is opposite to that on the right side. Therefore, finite changes recovered using such expressions may be underestimated and overestimated for positive and negative changes, respectively, or opposite depending on the curvature of the function. One approach for a more accurate recovery involves evaluating two changes (from the baseline to the perturbed state, and from the perturbed state to the baseline), where one will be underestimated and the other overestimated, and combining the two results. In this study, we apply this approach to make a finite-change correction to equations used in diffuse optics for recovering changes in optical properties from changes in measurands, such as light intensity changes.

Diffuse optics uses light to probe optically turbid media, which has many applications in biological tissues [1,2]. In particular, near-infrared spectroscopy (NIRS) is a non-invasive and safe technique for assessing tissue changes, e.g., in the brain, related to vital physiological parameters such as oxygenation and oxygen metabolism [3–6]. The clinical usefulness of NIRS has been demonstrated by a wide range of clinical applications and trials [7–9].

The Beer–Lambert law (BLL) establishes a linear relation between measured absorbance (*A*) and the concentration of chromophores (*C*) in the medium, making it widely used across many scientific fields [10–12]: A=εCd , where *d* is the distance light travelled and *ε* is the molar extinction coefficient of a chromophore. The BLL is the fundamental relation used for recovering concentrations *C*, but it is valid only in non-scattering media as it does not account for light losses due to scattering and assumes that *d* is independent of *C* [10–12].

In NIRS, light can penetrate deep into tissues (a few cm) and reemerge at the surface [3], which is enabled by tissue’s high scattering and low absorption in the NIR range [13–15]. This introduces light losses due to scattering and the mean optical pathlength *d* for the mean distance that light travels. The modified Beer-Lambert law (MBLL) is commonly used to recover changes (ΔC) from measured ΔA , under the assumption that the scattering properties remain constant in time and *d* is independent of ΔC [16]. The second assumption is not valid in scattering media since *d* decreases for an increase in *C*, as illustrated in [17]. Therefore, ΔA is non-linearly proportional to ΔC , and the MBLL is valid only for infinitesimal ΔC .

Among several measurement approaches available in diffuse optics (continuous-wave [6], frequency-domain [18], and multi-distance [19]), the time-resolved measurements provide the most information about light propagation, enabling data analysis methods based on other measurands in addition to *A* [20,21]. A laser emits pulses of light (up to ∼80 MHz repetition rate), and a single-photon detector coupled to a time-correlated single-photon counting card records photons arrival times (up to ∼10 ps resolution) [22], allowing to compute the distribution of times of flight of photons (DTOF) [21]. An analogue of the BLL in time domain was discussed in [23], where the shape of the temporal profile of a propagating light pulse was characterized. The common measurands used to recover changes in optical properties include changes in statistical moments of DTOFs [24,25], integrated intensity over time windows defined on the time axis of the DTOF, and ratios of such time windows [26]. These methods typically rely on perturbation theory and assume that changes in measurands are linearly proportional to changes in optical properties, which is valid only for infinitesimal changes. Although time-resolved measurements can be used to estimate absolute optical properties [21,27], and thereby their changes, this requires adequate integration time to acquire sufficient photon counts, which is challenging in monitoring dynamic changes.

In this study, we use perturbation theory to derive equations for sensitivity factors that relate ΔA and changes in statistical moments of DTOFs (mean time of flight $\Delta m_{1}$ and variance ΔV ) to infinitesimal changes in absorption $\left(\delta \mu_{a}\right)$ , reduced scattering $\left(\delta \mu_{s}^{\prime }\right)$ , and source-detector distance (δr) . This method for recovering changes in $\mu_{a}$ was developed by Liebert et al. [25] and has become well-established [28]. Next, we extend the perturbation-based equations to finite (including large) parameter changes (Δ*μ*_a_, $\Delta \mu_{s}^{\prime }$ , and Δ*r*). We consider homogeneous infinite medium (IM) and semi-infinite medium (SM) geometries [29]. The IM applies to measurements in which the source and detector are positioned inside a medium and far from boundaries compared to *r*. The SM applies to measurements on the surface of a medium, such as those obtained with optodes placed on tissues or other optically turbid medium.

The manuscript is organized as follows. In the Methods, we outline the derivations, which can be reproduced using the expressions provided in the Appendix, and present the derived equations for the sensitivity factors, along with equations for the MBLL that are valid for finite $\Delta \mu_{a}$ . In the Results, we verify the equations and show the improvement in parameter recovery, assess the impact of errors in the baseline optical properties or differential pathlength factor (DPF) , and evaluate the cross-talk due to changes in a second optical property. We discuss the implications and limitations of the presented equations and the derivation approach. Lastly, in the Appendix, we present closed-form solutions for $\Delta \mu_{a}$ for some of the derived equations.

Table 1 summarizes the main equations in this study, grouped according to their purpose in the derivation and recovery of optical property changes.

**Table 1.** Main equations grouped according to their purpose.

Description		Equations
Analytical solutions for the DTOF for IM and SM geometries		Eqs. (1) and (2)
DTOF moments in terms of optical properties		Eqs. (4)–(8)
Formula used to extend sensitivity factor equations to finite changes		Eq. (10)

Sensitivity factors for	infinitesimal δ*μ*_a_ and finite Δ*μ*_a_	Eqs. (11)–(22)
	infinitesimal $\delta \mu_{s}^{\prime }$ and finite $\Delta \mu_{s}^{\prime }$	Eqs. (23)–(34)
	finite Δ*r*	Eqs. (35)–(40)

Modified Beer-Lambert law (MBLL)		Eq. (41)
Differential pathlength factor (DPF) in terms of optical properties		Eq. (42)
MBLL for finite Δ*μ*_a_	for IM, and a closed-form solution for Δ*μ*_a_	Eqs. (44)–(45)
	for SM	Eq. (46)

Expressions substituted into Eq. (10) to derive sensitivity factors for finite Δ*μ*_a_ (Eqs. (17)–(22))		Eqs. (48)–(53)
Formulas for recovering finite Δ*μ*_a_ (Eqs. (17)–(22)), but without requiring nonlinear solvers		Eqs. (54)–(57)

## 2. Methods

### 2.1. Statistical moments of the distribution of times of flight of photons

We model the light source as a pulsed laser represented by a Dirac-delta light impulse. The source and detector are represented by two points in the medium. The photon fluence rate (φ) per incident photon (mm^−2^s^−1^) that reaches a detector located at a distance *r* from a light source is given by the following solution of the diffusion equation (DE) for an infinite medium (IM) [27]:

(1) $$\varphi (r,t)=\frac{c}{{\left(4\pi \text{Dct}\right)}^{3/2}}\text{exp}\left(-\frac{r^{2}}{4\text{Dct}}\right)\text{exp}(-\mu_{a}ct)$$

where *D* is the diffusion coefficient, *c* is the speed of light in the medium, and *t* is the arrival time of photons. We assume that *D* depends only on scattering, $D=1/(3\mu_{s}^{\prime })$ , because it was experimentally found that *D* is independent of $\mu_{a}$ in the diffusion regime [30,31]. Boundary conditions are not considered for IM.

The time-resolved reflectance R(r,t) , which represents the detected fraction of launched photons per unit area per unit time (mm^−2^ s^−1^) exiting the surface at a distance *r* from the light source, is given by the following solution of the DE for a semi-infinite medium (SM) [27] with the zero-boundary condition [32,33]. We made the approximation $\sqrt{r^{2}+{z_{0}}^{2}}\approx r$ , since typical measurements are carried out at relatively large source-detector separations $r\gg z_{0}$ , where $z_{0}=1/\mu_{s}^{\prime }$ is the effective depth at which the source is positioned.

(2) $$R(r,t)=\frac{z_{0}}{{\left(4\pi Dc\right)}^{3/2}t^{5/2}}\text{exp}\left(-\frac{r^{2}}{4\text{Dct}}\right)\text{exp}(-\mu_{a}ct)$$

R(r,t) models the DTOF measured in time-resolved diffuse optics experiments. The *k*-th order normalized non-central statistical moments (*m_k_*) of the DTOF are defined as:

(3) $$m_{k}=\left\langle t^{k}\right\rangle =\int_{-\infty }^{\infty }t^{k}\;\text{DTOF}(t)\;dt/\int_{-\infty }^{\infty }\text{DTOF}(t)\;dt$$

The zeroth-order moment (*m*_0_) is the integral of φ(r,t) or R(r,t) over time, which corresponds to the total number of detected photons $\left(N_{\text{tot}}\right)$ as a fraction of the number of incident photons. It can be used to calculate changes in attenuation $\Delta A=\text{ln}(N_{\text{tot},0}/N_{\text{tot}})=\text{ln}(I_{0}/I)$ , where $N_{\text{tot},0}$ and $N_{\text{tot},0}$ correspond to the reference state and after a parameter change, respectively, analogous to the reference (*I*_0_) and after a parameter change (*I*) light intensities. The first-order moment is the mean time of flight (*m*_1_). The second-order central moment is the variance (*V*) of the DTOF, which is related to non-central moments: $V=\left\langle {\left(t-\langle t\rangle \right)}^{2}\right\rangle =m_{2}-{m_{1}}^{2}$ . The third-order central moment $\left(m_{3,C}\right)$ is given by: $m_{3,C}=m_{3}-3m_{1}m_{2}+2{m_{1}}^{3}$ .

Equations (4)–(6) express the moments of the DTOF as functions of the medium’s optical properties. Noise increases rapidly with increasing moment order, and we previously found that moments beyond *V* are not useful in practice because of their high noise [34,35]. Therefore, we did not include $m_{3,C}$ in further analysis.

The equations for IM and SM (Eqs. (4)–(6)) differ only by dimensionless factors containing a ratio. Since this ratio is always less than 1, the values of moments in SM are smaller than those in IM for the same optical properties, and the difference decreases with increasing *r* and $\mu_{s}^{\prime }$ , and with decreasing $\mu_{a}$ .

(4) $$\begin{array}{llll} & m_{1,\text{IM}}=\frac{r}{2c\sqrt{D}{\mu_{a}}^{1/2}} & & m_{1,\text{SM}}=\frac{r}{2c\sqrt{D}{\mu_{a}}^{1/2}}\left(\frac{r\mu_{e}}{r\mu_{e}+1}\right) \end{array}$$

(5) $$\begin{array}{llll} & V_{\text{IM}}=\frac{r}{4c^{2}\sqrt{D}{\mu_{a}}^{3/2}} & & V_{\text{SM}}=\frac{r}{4c^{2}\sqrt{D}{\mu_{a}}^{3/2}}{\left(\frac{r\mu_{e}}{r\mu_{e}+1}\right)}^{2} \end{array}$$

(6) $$\begin{array}{llll} & m_{3,c,\text{IM}}=\frac{3r}{8c^{3}\sqrt{D}{\mu_{a}}^{5/2}} & & m_{3,c,\text{SM}}=\frac{3r}{8c^{3}\sqrt{D}{\mu_{a}}^{5/2}}\left(\frac{{\left(r\mu_{e}\right)}^{2}(r\mu_{e}+1/3)}{{\left(r\mu_{e}+1\right)}^{3}}\right) \end{array}$$

Rearranging these equations allows expressing the optical properties as a function of the measured moments (Eqs. (7) and (8)), which is an established method for estimating the optical properties of a homogeneous medium [24].

(7) $$\begin{array}{llll} & \mu_{a,\text{IM}}=\frac{m_{1}}{2cV} & & \mu_{a,\text{SM}}=\frac{m_{1}}{2cV}\left(\frac{{m_{1}}^{2}}{{m_{1}}^{2}+V}\right) \end{array}$$

(8) $$\begin{array}{llll} & \mu_{s,\text{IM}}^{\prime }=\frac{2c{m_{1}}^{3}}{3r^{2}V} & & \mu_{s,\text{SM}}^{\prime }=\frac{2c{m_{1}}^{3}}{3r^{2}V}\left(\frac{{m_{1}}^{2}+V}{{m_{1}}^{2}}\right) \end{array}$$

### 2.2. Derivation of sensitivity factors

Here, we outline the derivation of analytical equations for sensitivity factors (SF) that are valid for infinitesimal changes (denoted $SF^{\delta }$ ) and finite changes (denoted $SF^{\Delta }$ ). The measured values of *A*, *m*_1_, and *V* are influenced not only by the medium’s optical properties and the measurement geometry, but also by the laser, detector, and other instrumental factors. For the first four moments, these instrumental effects can be accounted for by measuring the instrument response function (IRF) and subtracting the moments of the IRF [36]. If the IRF remains constant during a measurement, the values of $\Delta A,\Delta m_{1},\Delta V$ , and $\Delta m_{3,C}$ depend solely on changes in optical properties of the medium, which forms the basis of a well-established method for recovering $\Delta \mu_{a}$ from changes in moments [25]. In this study, we additionally consider $\Delta \mu_{s}^{\prime }$ and Δr . The equations for $SF^{\delta }$ valid for infinitesimal $\delta \mu_{a},\delta \mu_{s}^{\prime }$ , or δr correspond to:

(9) $$S{F_{a}}^{\delta }=\frac{\partial M}{\partial \mu_{a}}\;\;\;S{F_{s}}^{\delta }=\frac{\partial M}{\partial \mu_{s}^{\prime }}\;\;\;\;S{F_{r}}^{\delta }=\frac{\partial M}{\partial r}$$

where *M* represents the considered measurand (*A*, *m*_1_, or *V*). SF are referred to as the mean partial pathlength (*MPP*) for ΔA , the mean time of flight sensitivity factor (MTSF) for $\Delta m_{1}$ , and the variance sensitivity factor (*VSF*) for ΔV . Throughout this study, the subscripts denote absorption (a), scattering (s), and source-detector distance (*r*). To derive equations for $\text{MTS}F^{\delta }$ and $VSF^{\delta }$ based on perturbation theory, we differentiated the expressions for *m*_1_ and *V*, respectively, given in the previous section, with respect to $\mu_{a},\mu_{s}^{\prime }$ , or *r*. To derive an equation for $MPP^{\delta }$ , we differentiated $A=\text{ln}(1/m_{0})$ , as defined in the previous section, with respect to $\mu_{a},\mu_{s}^{\prime }$ , or *r*.

Using $\Delta \mu_{a}$ as an example, the method based on sensitivity factors for recovery of $\Delta \mu_{a}$ uses the relation $\Delta \mu_{a}=\Delta M\;/\;SF_{a}$ . To extend the validity of $S{F_{a}}^{\delta }$ to finite changes, i.e., derive $S{F_{a}}^{\Delta }$ , we implement the following approach. As mentioned in the Introduction, we recover two changes (relative to baseline, and relative to perturbed state), where one is overestimated and the other is underestimated, and combine the two results. Accurate $\Delta \mu_{a}$ can be recovered using the following formula:

(10) $$\Delta \mu_{a}=\frac{\Delta \mu_{a,1}}{2}+\frac{\Delta \mu_{a,2}}{2}=\frac{\Delta M}{2\;SF_{a}(\mu_{a})}+\frac{\Delta M}{2\;SF_{a}({\mu_{a}}^{*})}=\Delta M\left(\frac{SF_{a}(\mu_{a})+SF_{a}({\mu_{a}}^{*})}{2\;SF_{a}(\mu_{a})\;SF_{a}({\mu_{a}}^{*})}\right)=\Delta M\frac{1}{S{F_{a}}^{\Delta }}$$

where ${\mu_{a}}^{*}=\mu_{a}+\Delta \mu_{a}$ , and $SF_{a}(\mu_{a})$ and $SF_{a}({\mu_{a}}^{*})$ are the sensitivity factor expressions for the state before and after $\Delta \mu_{a}$ , respectively. $\Delta \mu_{a,1}$ is the change recovered using $SF_{a}(\mu_{a})$ , and $\Delta \mu_{a,2}$ is the change recovered using $SF_{a}({\mu_{a}}^{*})$ . As shown in Eq. (10), we can derive an expression for $S{F_{a}}^{\Delta }$ such that $\Delta \mu_{a}$ can be recovered using the relation $\Delta \mu_{a}=\Delta M\;/\;S{F_{a}}^{\Delta }$ . All expressions for $SF_{a}(\mu_{a})$ and $SF_{a}({\mu_{a}}^{*})$ , which we substituted into Eq. (10) to derive the $S{F_{a}}^{\Delta }$ equations, are provided in the Appendix, and they were obtained using the following approach.

To derive $MP{P_{a}}^{\Delta }$ for IM and SM, we substituted the corresponding $MP{P_{a}}^{\delta }(\mu_{a})$ and $MP{P_{a}}^{\delta }(\mu_{a}={\mu_{a}}^{*})$ for $SF_{a}(\mu_{a})$ and $SF_{a}({\mu_{a}}^{*})$ , respectively, in Eq. (10). The same approach was used to derive all $S{F_{s}}^{\Delta }$ expressions, i.e., $MP{P_{s}}^{\Delta },\text{MTS}{F_{s}}^{\Delta }$ , and $VS{F_{s}}^{\Delta }$ , for IM and SM, by substituting the corresponding $S{F_{s}}^{\delta }(\mu_{s}^{\prime })$ and $S{F_{s}}^{\delta }(\mu_{s}^{\prime }=\mu_{s}^{\prime }*)$ into Eq. (10).

In contrast, to derive $\text{MTS}{F_{a}}^{\Delta }$ and $VS{F_{a}}^{\Delta }$ for IM, the expressions for $SF_{a}(\mu_{a})$ and $SF_{a}({\mu_{a}}^{*})$ contain both $\mu_{a}$ and ${\mu_{a}}^{*}$ . This makes the magnitudes of overestimation and underestimation in $\Delta \mu_{a,1}$ and $\Delta \mu_{a,2}$ equal, so that they cancel in Eq. (10). Next, to derive $\text{MTS}{F_{a}}^{\Delta }$ and $VS{F_{a}}^{\Delta }$ for SM, we use their equations for IM and the relation between the equations for IM and SM for moments (Eqs. (4)–(6)). The expressions in the Appendix provide the information needed to reproduce the derivations.

*m*_1_ and *V* in IM are proportional to *r* (Eqs. (4) and (5)), and hence $\text{MTS}{F_{r}}^{\delta }$ and $VS{F_{r}}^{\delta }$ in IM are valid for any magnitude Δr . In SM, *m*_1_ and *V* are nearly proportional to *r*. Also, *A* in IM and SM is nearly proportional to *r*, which results from $A=\text{ln}(1/m_{0})$ , where *m*_0_ is the integral of Eqs. (1) and (2) for IM and SM, respectively. Therefore, we derived the $S{F_{r}}^{\Delta }$ equations by obtaining $S{F_{r}}^{\delta }$ (differentiating the moments with respect to *r*) and replacing *r* with $r_{\text{av}}=r+\Delta r/2$ , which is the mean of the initial and changed *r*.

### 2.3. Sensitivity factors for $\Delta \mu_{a}$

Equations (11)–(13) express $S{F_{a}}^{\delta }$ for IM in terms of optical properties or moments, whereas Eqs. (14)–(16) express them for SM, in terms of moments. The values of $S{F_{a}}^{\delta }$ decrease as $\mu_{a}$ increases, hence the magnitude of the measured changes in moments grows less with increasing $\Delta \mu_{a}$ , leading to an underestimation in the recovered $\Delta \mu_{a}$ . The equations for IM and SM (Eqs. (11)–(13)) differ by the same dimensionless factors as in the equations for moments (Eqs. (4)–(6)). This similarity is used to derive the $\text{MTS}{F_{a}}^{\Delta }$ and $VS{F_{a}}^{\Delta }$ equations for SM, as explained in the previous section.

(11) $$\begin{array}{llll} & MP{P_{a,\text{IM}}}^{\delta }=\frac{\partial A}{\partial \mu_{a}}=\frac{r\sqrt{3\mu_{s}^{\prime }}}{2\sqrt{\mu_{a}}} & & MP{P_{a,\text{SM}}}^{\delta }=\frac{r\sqrt{3\mu_{s}^{\prime }}}{2\sqrt{\mu_{a}}}\left(\frac{r\mu_{e}}{r\mu_{e}+1}\right) \end{array}$$

(12) $$\begin{array}{llll} & \text{MTS}{F_{a,\text{IM}}}^{\delta }=\frac{\partial m_{1}}{\partial \mu_{a}}=-\frac{1}{4c}\frac{r\sqrt{3\mu_{s}^{\prime }}}{{\mu_{a}}^{3/2}} & & \text{MTS}{F_{a,\text{SM}}}^{\delta }=-\frac{1}{4c}\frac{r\sqrt{3\mu_{s}^{\prime }}}{{\mu_{a}}^{3/2}}{\left(\frac{r\mu_{e}}{r\mu_{e}+1}\right)}^{2} \end{array}$$

(13) $$\begin{array}{llll} & VS{F_{a,\text{IM}}}^{\delta }=\frac{\partial V}{\partial \mu_{a}}=-\frac{3}{8c^{2}}\frac{r\sqrt{3\mu_{s}^{\prime }}}{{\mu_{a}}^{5/2}} & & VS{F_{a,\text{SM}}}^{\delta }=-\frac{3}{8c^{2}}\frac{r\sqrt{3\mu_{s}^{\prime }}}{{\mu_{a}}^{5/2}}\left(\frac{{\left(r\mu_{e}\right)}^{2}(r\mu_{e}+1/3)}{{\left(r\mu_{e}+1\right)}^{3}}\right) \end{array}$$

(14) $$\begin{array}{llll} & MP{P_{a,\text{IM}}}^{\delta }=cm_{1} & & MP{P_{a,\text{SM}}}^{\delta }=cm_{1} \end{array}$$

(15) $$\begin{array}{llll} & \text{MTS}{F_{a,\text{IM}}}^{\delta }=-cV & & \text{MTS}{F_{a,\text{SM}}}^{\delta }=-cV \end{array}$$

(16) $$\begin{array}{llll} & VS{F_{a,\text{IM}}}^{\delta }=-\frac{3cV^{2}}{m_{1}}=-cm_{3,c} & & VS{F_{a,\text{SM}}}^{\delta }=-\frac{3cV^{2}}{m_{1}}\left(\frac{3{m_{1}}^{2}+V}{3{m_{1}}^{2}}\right)=-cm_{3,c} \end{array}$$

Equations (17)–(19) express $S{F_{a}}^{\Delta }$ in terms of optical properties, while Eqs. (20)–(22) express them in SM, where ${\mu_{a}}^{*}=\mu_{a}+\Delta \mu_{a}$ , and $\mu_{e}=\sqrt{3\mu_{a}\mu_{s}^{\prime }}$ , and ${\mu_{e}}^{*}=\sqrt{3{\mu_{a}}^{*}\mu_{s}^{\prime }}$ , and ${m_{1}}^{*}=m_{1}+\Delta m_{1}$ . These equations for finite changes (Eqs. (17)–(22)) simplify to those for infinitesimal changes (Eqs. (11)–(16)) as $\Delta \mu_{a}$ approaches 0.

(17) $$MP{P_{a,\text{IM}}}^{\Delta }=\frac{\Delta A}{\Delta \mu_{a}}=\frac{r\sqrt{3\mu_{s}^{\prime }}}{\sqrt{\mu_{a}}+\sqrt{{\mu_{a}}^{*}}}=cm_{1}\left(\frac{2{m_{1}}^{*}}{m_{1}+{m_{1}}^{*}}\right)$$

(18) $$\text{MTS}{F_{a,\text{IM}}}^{\Delta }=\frac{\Delta m_{1}}{\Delta \mu_{a}}=-\frac{r\sqrt{3\mu_{s}^{\prime }}}{2c\sqrt{\mu_{a}{\mu_{a}}^{*}}\left(\sqrt{\mu_{a}}+\sqrt{{\mu_{a}}^{*}}\right)}=cm_{1}\left(\frac{2{m_{1}}^{*}}{m_{1}+{m_{1}}^{*}}\right)\left(\frac{\Delta A}{\Delta m_{1}}\right)$$

(19) $$VS{F_{a,\text{IM}}}^{\Delta }=\frac{\Delta V}{\Delta \mu_{a}}=-\frac{3r\sqrt{3\mu_{s}^{\prime }}}{4c^{2}\mu_{a}\mu_{a}^{*}\left(\sqrt{\mu_{a}}+\sqrt{\mu_{a}^{*}}\right)}=-6m_{1}\left(\frac{m_{1}^{*}}{m_{1}+m_{1}^{*}}\right){\left(\frac{\Delta A}{\Delta m_{1}}\right)}^{2}$$

(20) $$MP{P_{a,\text{SM}}}^{\Delta }=\frac{3r^{2}\mu_{s}^{\prime }}{r\sqrt{3\mu_{s}^{\prime }}\left(\sqrt{\mu_{a}}+\sqrt{{\mu_{a}}^{*}}\right)+2}$$

(21) $$\text{MTS}{F_{a,\text{SM}}}^{\Delta }=-\frac{r\sqrt{3\mu_{s}^{\prime }}}{c\sqrt{\mu_{a}{\mu_{a}}^{*}}\left(\sqrt{\mu_{a}}+\sqrt{{\mu_{a}}^{*}}\right)}{\left[{\left(\frac{r\mu_{e}+1}{r\mu_{e}}\right)}^{2}+{\left(\frac{r{\mu_{e}}^{*}+1}{r{\mu_{e}}^{*}}\right)}^{2}\right]}^{-1}$$

(22) $$VS{F_{a,\text{SM}}}^{\Delta }=-\frac{3r\sqrt{3\mu_{s}^{\prime }}}{2c^{2}\mu_{a}{\mu_{a}}^{*}\left(\sqrt{\mu_{a}}+\sqrt{{\mu_{a}}^{*}}\right)}{\left[\frac{{\left(r\mu_{e}+1\right)}^{3}}{r^{2}{\mu_{e}}^{2}(r\mu_{e}+1/3)}+\frac{{\left(r{\mu_{e}}^{*}+1\right)}^{3}}{r^{2}{\mu_{e}}^{*2}(r{\mu_{e}}^{*}+1/3)}\right]}^{-1}$$

### 2.4 Sensitivity factors for $\Delta \mu_{s}^{\prime }$

**Fig. 1.**
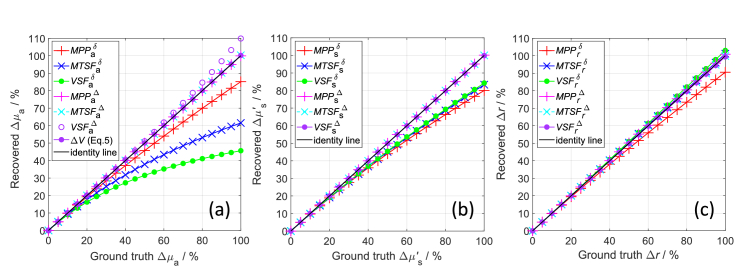
Analysis of the validity of the sensitivity factor equations for recovery of (a) $\Delta \mu_{a}$ using Eqs. (11)–(22), (b) $\Delta \mu_{s}^{\prime }$ using Eqs. (23)–(34), and (c) Δr using Eqs. (35)–(40). Results are shown for $SF^{\delta }$ (valid for infinitesimal changes; red, blue, and green curves) and $SF^{\Delta }$ (valid for finite changes; magenta, cyan, and purple curves).

Equations (23)–(25) express $S{F_{s}}^{\delta }$ in terms of optical properties, while Eqs. (26)–(28) express them in terms of moments, where $\mu_{s}^{\prime *}=\mu_{s}^{\prime }+\Delta \mu_{s}^{\prime }$ , and ${\mu_{e}}^{*}=\sqrt{3\mu_{a}\mu_{s}^{\prime *}}$ , and $V^{*}=V+\Delta V$ . $S{F_{s}}^{\delta }$ are proportional to $\mu_{s}^{\prime -1/2}$ , similar as $MP{P_{a}}^{\delta }$ is proportional to ${\mu_{a}}^{-1/2}$ , except $MP{P_{s}}^{\delta }$ has an additional term containing $1/\mu_{s}^{\prime }$ . Therefore, the finite-change correction will have a similar improvement for $\Delta \mu_{s}^{\prime }$ recovery as for $\Delta \mu_{a}$ recovery using $MPP_{a}$ , as shown in Fig. 1.

(23) $$\begin{array}{llll} & MP{P_{s,\text{IM}}}^{\delta }=\frac{\partial A}{\partial \mu_{s}^{\prime }}=\frac{r\sqrt{3\mu_{a}}}{2\sqrt{\mu_{s}^{\prime }}}-\frac{1}{\mu_{s}^{\prime }} & & MP{P_{s,\text{SM}}}^{\delta }=\frac{r\mu_{e}+1}{2\mu_{s}^{\prime }}+\frac{1}{2\mu_{s}^{\prime }(r\mu_{e}+1)} \end{array}$$

(24) $$\begin{array}{llll} & \text{MTS}{F_{s,\text{IM}}}^{\delta }=\frac{\partial m_{1}}{\partial \mu_{s}^{\prime }}=\frac{r\sqrt{3}}{4c\sqrt{\mu_{a}\mu_{s}^{\prime }}} & & \text{MTS}{F_{s,\text{SM}}}^{\delta }=\frac{r\sqrt{3}}{4c\sqrt{\mu_{a}\mu_{s}^{\prime }}}\left(\frac{r\mu_{e}(r\mu_{e}+2)}{{\left(r\mu_{e}+1\right)}^{2}}\right) \end{array}$$

(25) $$\begin{array}{llll} & VS{F_{s,\text{IM}}}^{\delta }=\frac{\partial V}{\partial \mu_{s}^{\prime }}=\frac{r\sqrt{3}}{8c^{2}{\mu_{a}}^{3/2}\sqrt{\mu_{s}^{\prime }}} & & VS{F_{s,\text{SM}}}^{\delta }=\frac{r\sqrt{3}}{8c^{2}{\mu_{a}}^{3/2}\sqrt{\mu_{s}^{\prime }}}\left(\frac{{\left(r\mu_{e}\right)}^{2}(r\mu_{e}+3)}{{\left(r\mu_{e}+1\right)}^{3}}\right) \end{array}$$

(26) $$\begin{array}{llll} & MP{P_{s,\text{IM}}}^{\delta }=\frac{3r^{2}}{4cm_{1}}\left(\frac{{m_{1}}^{2}-2V}{{m_{1}}^{2}}\right) & & MP{P_{s,\text{SM}}}^{\delta }=\frac{3r^{2}}{4cm_{1}}\left(1+\frac{V^{2}}{{\left({m_{1}}^{2}+V\right)}^{2}}\right) \end{array}$$

(27) $$\begin{array}{llll} & \text{MTS}{F_{s,\text{IM}}}^{\delta }=\frac{3r^{2}}{4cm_{1}}\left(\frac{V}{m_{1}}\right) & & \text{MTS}{F_{s,\text{SM}}}^{\delta }=\frac{3r^{2}V}{4c}\left(\frac{{m_{1}}^{2}+2V}{{\left({m_{1}}^{2}+V\right)}^{2}}\right) \end{array}$$

(28) $$\begin{array}{llll} & VS{F_{s,\text{IM}}}^{\delta }=\frac{3r^{2}}{4cm_{1}}{\left(\frac{V}{m_{1}}\right)}^{2} & & VS{F_{s,\text{SM}}}^{\delta }=\frac{3r^{2}}{4cm_{1}}\left(\frac{V^{2}}{m_{1}}\right)\left(\frac{{m_{1}}^{2}+3V}{{\left({m_{1}}^{2}+V\right)}^{2}}\right) \end{array}$$

Equations (29)–(31) express $S{F_{s}}^{\Delta }$ for IM in terms of optical properties or moments, whereas Eqs. (32)–(34) express $S{F_{s}}^{\Delta }$ for SM. These equations for finite changes (Eqs. (29)–(34)) simplify to those for infinitesimal changes (Eqs. (23)–(28)) as $\Delta \mu_{s}^{\prime }$ approaches 0.

(29) $$MP{P_{s,\text{IM}}}^{\Delta }=\frac{\Delta A}{\Delta \mu_{s}^{\prime }}={\left[\frac{\mu_{s}^{\prime }}{r\mu_{e}-2}+\frac{\mu_{s}^{\prime *}}{r{\mu_{e}}^{*}-2}\right]}^{-1}=\frac{3r^{2}}{2c}\left[\frac{\left({m_{1}}^{2}-2V_{1})({m_{1}}^{*2}-2{V_{1}}^{*}\right)}{{m_{1}}^{*3}({m_{1}}^{2}-2V_{1})+{m_{1}}^{3}({m_{1}}^{*2}-2{V_{1}}^{*})}\right]$$

(30) $$\text{MTS}{F_{s,\text{IM}}}^{\Delta }=\frac{\Delta m_{1}}{\Delta \mu_{s}^{\prime }}=\frac{r\sqrt{3}}{2c\sqrt{\mu_{a}}\left(\sqrt{\mu_{s}^{\prime }}+\sqrt{\mu_{s}^{\prime *}}\right)}=\frac{3r^{2}}{2c}{\left(\frac{{m_{1}}^{2}}{V}+\frac{{m_{1}}^{*2}}{V^{*}}\right)}^{-1}$$

(31) $$VS{F_{s,\text{IM}}}^{\Delta }=\frac{\Delta V}{\Delta \mu_{s}^{\prime }}=\frac{r\sqrt{3}}{4c^{2}{\mu_{a}}^{3/2}\left(\sqrt{\mu_{s}^{\prime }}+\sqrt{\mu_{s}^{\prime *}}\right)}=\frac{3r^{2}}{2c}{\left(\frac{{m_{1}}^{3}}{V^{2}}+\frac{{m_{1}}^{*3}}{{V^{*}}^{2}}\right)}^{-1}$$

(32) $$MP{P_{s,\text{SM}}}^{\Delta }={\left[\frac{\mu_{s}^{\prime }(r\mu_{e}+1)}{{\left(r\mu_{e}+1\right)}^{2}+1}+\frac{\mu_{s}^{\prime *}(r{\mu_{e}}^{*}+1)}{{\left(r{\mu_{e}}^{*}+1\right)}^{2}+1}\right]}^{-1}$$

(33) $$\text{MTS}{F_{s,\text{SM}}}^{\Delta }=\frac{r\sqrt{3}}{c\sqrt{\mu_{a}}\left(\sqrt{\mu_{s}^{\prime }}+\sqrt{\mu_{s}^{\prime *}}\right)}{\left[\frac{{\left(r\mu_{e}+1\right)}^{2}}{r\mu_{e}(r\mu_{e}+2)}+\frac{{\left(r{\mu_{e}}^{*}+1\right)}^{2}}{r{\mu_{e}}^{*}(r{\mu_{e}}^{*}+2)}\right]}^{-1}$$

(34) $$VS{F_{s,\text{SM}}}^{\Delta }=\frac{r\sqrt{3}}{2c^{2}{\mu_{a}}^{3/2}\left(\sqrt{\mu_{s}^{\prime }}+\sqrt{\mu_{s}^{\prime *}}\right)}{\left[\frac{{\left(r\mu_{e}+1\right)}^{3}}{r^{2}{\mu_{e}}^{2}(r\mu_{e}+3)}+\frac{{\left(r{\mu_{e}}^{*}+1\right)}^{3}}{r^{2}{\mu_{e}}^{*2}(r{\mu_{e}}^{*}+3)}\right]}^{-1}$$

### 2.5 Sensitivity factors for Δr

Equations (35)–(37) express $S{F_{r}}^{\Delta }$ in terms of optical properties, while Eqs. (38)–(40) express them in terms of moments. The change in interoptode distance Δr is a known parameter during measurements and hence it is not meaningful to recover it from the measured data. However, we present $S{F_{r}}^{\Delta }$ equations because they can be used to recover absolute optical properties from multi-distance measurements [37]. In such data analyses, Δr is a known parameter, $S{F_{r}}^{\Delta }$ is unknown, which is a function of $\mu_{a}$ and $\mu_{s}^{\prime }$ , and the changes in moments are the measurands. In contrast, the sensitivity factor approach is typically used to recover the changed parameter rather than the baseline optical properties. The variable $r_{\text{av}}$ is the average of the initial and changed values of *r*, given by $r_{\text{av}}=r+\Delta r/2$ .

$\text{MTS}{F_{r}}^{\Delta }$ and $VS{F_{r}}^{\Delta }$ in IM (Eqs. (36) and (37)) do not depend on *r*, consistent with the equations for *m*_1_ and *V* (Eqs. (4) and (5)).

(35) $$\begin{array}{llll} & MP{P_{r,\text{IM}}}^{\Delta }=\mu_{e}+\frac{1}{r_{\text{av}}} & & MP{P_{r,\text{SM}}}^{\Delta }=\left(\mu_{e}+\frac{2}{r_{\text{av}}}-\frac{3}{{r_{\text{av}}}^{3}{\mu_{e}}^{2}}\right)\left(\frac{{r_{\text{av}}}^{2}{\mu_{e}}^{2}}{{r_{\text{av}}}^{2}{\mu_{e}}^{2}-1}\right) \end{array}$$

(36) $$\begin{array}{llll} & \text{MTS}{F_{r,\text{IM}}}^{\Delta }=\frac{1}{2c\sqrt{D}}\frac{1}{\sqrt{\mu_{a}}} & & \text{MTS}{F_{r,\text{SM}}}^{\Delta }=\frac{1}{2c\sqrt{D}}\frac{1}{\sqrt{\mu_{a}}}\left(\frac{r_{\text{av}}\mu_{e}(r_{\text{av}}\mu_{e}+2)}{{\left(r_{\text{av}}\mu_{e}+1\right)}^{2}}\right) \end{array}$$

(37) $$\begin{array}{llll} & VS{F_{r,\text{IM}}}^{\Delta }=\frac{1}{4c^{2}\sqrt{D}}\frac{1}{{\mu_{a}}^{3/2}} & & VS{F_{r,\text{SM}}}^{\Delta }=\frac{1}{4c^{2}\sqrt{D}}\frac{1}{{\mu_{a}}^{3/2}}\left(\frac{{\left(r\mu_{e}\right)}^{2}(r_{\text{av}}\mu_{e}+3)}{{\left(r_{\text{av}}\mu_{e}+1\right)}^{3}}\right) \end{array}$$

(38) $$\begin{array}{llll} & MP{P_{r,\text{IM}}}^{\Delta }=\frac{{m_{1}}^{2}}{rV}+\frac{1}{r_{\text{av}}} & & MP{P_{r,\text{SM}}}^{\Delta }=\frac{{m_{1}}^{2}}{r\;V}\left(\frac{{m_{1}}^{2}}{{m_{1}}^{2}+V^{*}}\right)+\frac{3}{r_{\text{av}}} \end{array}$$

(39) $$\begin{array}{llll} & \text{MTS}{F_{r,\text{IM}}}^{\Delta }=\frac{m_{1}}{r} & & \text{MTS}{F_{r,\text{SM}}}^{\Delta }=\frac{m_{1}}{r}\left(\frac{{m_{1}}^{2}+2V}{{m_{1}}^{2}+V}\right) \end{array}$$

(40) $$\begin{array}{llll} & VS{F_{r,\text{IM}}}^{\Delta }=\frac{V}{r} & & VS{F_{r,\text{SM}}}^{\Delta }=\frac{V}{r}\left(\frac{{m_{1}}^{2}+3V}{{m_{1}}^{2}+V}\right) \end{array}$$

### 2.6. Modified Beer–Lambert law for finite $\Delta \mu_{a}$

The equation for $MP{P_{a}}^{\delta }$ (Eq. (11)), which relates ΔA to an infinitesimal $\delta \mu_{a}$ , is analogous to the modified Beer–Lambert law (MBLL), but expressed in terms of optical properties rather than the differential pathlength factor (DPF) . Here, we derive analytical equations for the MBLL that are valid for finite $\Delta \mu_{a}$ by expressing $MP{P_{a}}^{\Delta }$ in terms of *DPF*. The MBLL, which is valid for infinitesimal $\delta \mu_{a}$ , is [16]:

(41) $$A=-\text{log}\left(\frac{I}{I_{0}}\right)=-\text{log}\left(\frac{N_{\text{tot}}}{N_{\text{tot},0}}\right)=r\;\text{DPF}\;\mu_{a}+G$$

where *DPF* (dimensionless) is a scaling factor that relates *r* to the mean pathlength travelled by photons [38]. *G* is an unknown geometry-dependent factor that accounts for light losses due to scattering and measurement geometry. Assuming *G* remains constant during a measurement, Eq. (41) allows recovering changes in absorption using the relation: $\Delta A=r\;\text{DPF}\;\Delta \mu_{a}$ . By definition, $\text{DPF}=cm_{1}/r=MP{P_{a}}^{\delta }/r$ . Using the equation for $MP{P_{a}}^{\delta }$ (Eq. (11)), we express *DPF* in terms of optical properties for IM and SM:

(42) $$DPF_{\text{IM}}=\frac{cm_{1}}{r}=\frac{MP{P_{a,\text{IM}}}^{\delta }}{r}=\frac{\sqrt{3\mu_{s}^{\prime }}}{2\sqrt{\mu_{a}}}\;DPF_{\text{SM}}=\frac{MP{P_{a,\text{SM}}}^{\delta }}{r}=\frac{\sqrt{3\mu_{s}^{\prime }}}{2\sqrt{\mu_{a}}}\left(\frac{r\sqrt{3\mu_{a}\mu_{s}^{\prime }}}{r\sqrt{3\mu_{a}\mu_{s}^{\prime }}+1}\right)$$

As expected, *DPF* increases with $\mu_{s}^{\prime }$ and decreases with $\mu_{a}$ , whereas the MBLL assumes that *DPF* remains constant after $\Delta \mu_{a}$ . *DPF* is independent of *r* for the IM, but there is a small dependence on *r* for the SM. To derive analytical equations for the MBLL that are valid for finite $\Delta \mu_{a}$ in IM and SM, we express $MP{P_{a}}^{\Delta }$ (Eq. (17)) in terms of $MP{P_{a}}^{\delta }$ (Eq. (11)). For the IM geometry, the relation is:

(43) $$MP{P_{a,\text{IM}}}^{\Delta }=MP{P_{a,\text{IM}}}^{\delta }\left(\frac{2}{1+\sqrt{1+\Delta \mu_{a}/\mu_{a}}}\right)$$

We substitute the following two definitions: $MP{P_{a}}^{\Delta }=\Delta A/\Delta \mu_{a}$ and $MP{P_{a}}^{\delta }=r\;\text{DPF}$ . The resulting equation for the MBLL that is valid for finite $\Delta \mu_{a,\text{IM}}$ in the IM is:

(44) $$\Delta A=r\;\text{DPF}\left(\frac{2}{1+\sqrt{1+\Delta \mu_{a,\text{IM}}/\mu_{a}}}\right)\Delta \mu_{a,\text{IM}}$$

The parameter $\Delta \mu_{a,\text{IM}}$ in Eq. (44) can be isolated to simplify its recovery from the measured ΔA :

(45) $$\Delta \mu_{a,\text{IM}}=\frac{\Delta A}{r\;\text{DPF}}+{\left(\frac{\Delta A}{2r\;\text{DPF}\sqrt{\mu_{a}}}\right)}^{2}$$

The first term is the same as the MBLL (Eq. (41)). The second term, which contains $\Delta A^{2}$ , is an order of magnitude smaller than the first term for a 10% $\Delta \mu_{a}$ and typical tissue optical properties *μ*_a_ = 0.1 cm^−1^ and $\mu_{s}^{\prime }=10\;{\text{cm}}^{-1}$ . Equation (45) is the same as Eq. (54) in the Appendix but expressed in terms of *DPF* given in Eq. (42) rather than solely in terms of optical properties.

Repeating the above approach for the SM geometry yields the following equation for the MBLL that is valid for finite $\Delta \mu_{a,\text{SM}}$ in the SM:

(46) $$\Delta A=\text{r DPF}\left(\frac{2r\sqrt{3\mu_{s}^{\prime }\;\mu_{a}}+2}{r\sqrt{3\mu_{s}^{\prime }}\left(\sqrt{\mu_{a}}+\sqrt{\mu_{a}+\Delta \mu_{a,\text{SM}}}\right)+2}\right)\;\Delta \mu_{a,\text{SM}}$$

**Fig. 2.**
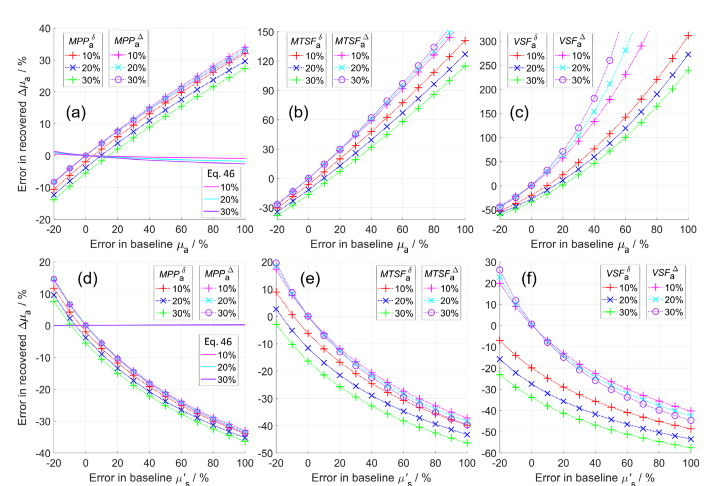
Errors in the recovered $\Delta \mu_{a}$ due to errors in the assumed baseline values of $\mu_{a}$ (top row) and $\mu_{s}^{\prime }$ (bottom row) using sensitivity factors for ΔA (a, d), $\Delta m_{1}$ (b, e), or ΔV (c, f). Legends show the magnitude of $\Delta \mu_{a}$ relative to baseline. Accurate DPF value was used in Eq. (46).

**Fig. 3.**
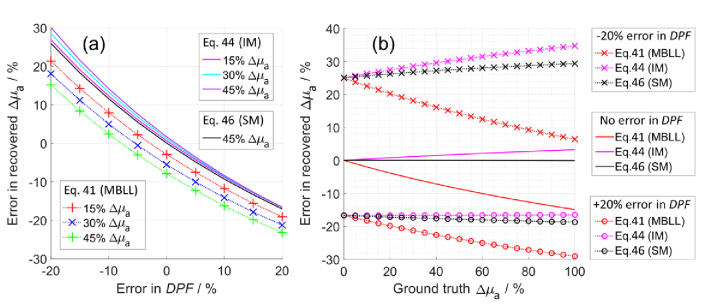
Errors in the recovered $\Delta \mu_{a}$ due to errors in the assumed DPF value, shown for a range of errors in DPF (a) and for a range of $\Delta \mu_{a}$ values (b). Equations (44) and (46) are the derived finite-change equations of the modified Beer–Lambert law (Eq. (41)) for infinite medium (IM) and semi-infinite medium (SM) geometries, respectively.

A closed-form solution for $\Delta \mu_{a,\text{SM}}$ could not be obtained from Eq. (46), so nonlinear solvers should be used to recover $\Delta \mu_{a,\text{SM}}$ . Alternatively, the closed-form equation for $\Delta \mu_{a,\text{IM}}$ (Eq. (45)) can be used as a good approximation for recovering $\Delta \mu_{a,\text{SM}}$ , as later shown in Fig. 3. Equations (44) and (46) contain the baseline values of $\mu_{a}$ and $\mu_{s}^{\prime }$ . However, errors in these parameters lead to negligible errors in the recovered $\Delta \mu_{a}$ , as later shown in Fig. 2.

The MBLL is typically used for recovering changes in concentrations (ΔC) of chromophores, rather than $\Delta \mu_{a}$ . In NIRS, the main chromophores of interest are oxyhemoglobin $\left(C_{\text{HbO}2}\right)$ and deoxyhemoglobin $\left(C_{\text{Hb}}\right)$ . Their concentration changes can be recovered using the relation: $\Delta \mu_{a}(\lambda )=\;\varepsilon_{\text{HbO}2}(\lambda )\;\Delta C_{\text{HbO}2}+\;\varepsilon_{\text{Hb}}(\lambda )\;\;\Delta C_{\text{Hb}}$ , where $\;\varepsilon_{\text{HbO}2}$ and $\;\varepsilon_{\text{Hb}}$ are the molar extinction coefficients of oxy- and deoxyhemoglobin, respectively, at wavelength *λ* [39]. Measurements at additional wavelengths enable recovery of changes in more chromophores, such as cytochrome-c-oxidase, which provides information on the rate of cellular oxygen consumption [4].

## 3. Results

All results presented below were obtained for the SM geometry, and results for the IM geometry have negligible differences (not shown). The baseline optical properties of the medium are $\mu_{a}=0.1\;{\text{cm}}^{-1}$ , $\mu_{s}^{\prime }=10\;{\text{cm}}^{-1}$ , n=1.4 , and *r* = 3 cm, unless stated otherwise. All results are presented as percentages relative to these values.

### 3.1. Verification of equations

To verify the sensitivity factor equations, we generated DTOFs using Eq. (2) for the baseline parameters and after parameter changes, and calculated $\Delta A,\Delta m_{1}$ , and ΔV from these DTOFs over the range from 0 to 7 ns. We recovered the parameter changes by solving the equations in Tables 3 to 7 using the *fsolve* function in MATLAB. Figure 1 presents the recovered $\Delta \mu_{a},\Delta \mu_{s}^{\prime }$ , and Δr .

For an increase in $\mu_{a}$ (Fig. 1(a)), the recovered $\Delta \mu_{a}$ is underestimated when using $S{F_{a}}^{\delta }$ (Eqs. (11)–(13)), and the error increases with the magnitude of the change. The values of $S{F_{a}}^{\delta }$ decreases with increasing $\mu_{a}$ , whereas they are assumed constant in the recovery of $\Delta \mu_{a}$ . In contrast, $S{F_{a}}^{\Delta }$ (Eqs. (17)–(19)), is a function of $\Delta \mu_{a}$ and allows accurate recovery of $\Delta \mu_{a}$ of any magnitude, except for a slight deviation when using $VS{F_{a}}^{\Delta }$ , resulting in an error of approximately 2% (or 10%) for a 50% (or 100%) increase in $\mu_{a}$ . For $\Delta \mu_{a}$ larger than 100%, the error grows rapidly and Eq. (5) may be used instead of $VS{F_{a}}^{\Delta }$ . We found that these percentage errors have only a small dependence on the baseline optical properties and *r*. The equation for *V* (Eq. (5)) is accurate for any magnitude of $\Delta \mu_{a}$ , but it is challenging to invert it for $\Delta \mu_{a}$ and it is not well suited for stable numerical solution.

For an increase in $\mu_{s}^{\prime }$ (Fig. 1(b)), the percentage errors in the recovered $\Delta \mu_{s}^{\prime }$ are similar to the percentage errors in the recovered $\Delta \mu_{a}$ using $MP{P_{a}}^{\delta }$ (Fig. 1(a)), as expected since $S{F_{s}}^{\delta }$ are proportional to $\mu_{s}^{\prime -1/2}$ and $MP{P_{a}}^{\delta }$ is proportional to ${\mu_{a}}^{-1/2}$ . $MP{P_{s}}^{\delta }$ has an additional term containing $\mu_{s}^{\prime -1}$ , but its value is much smaller than the term containing $\mu_{s}^{\prime -1/2}$ .

The results presented in Fig. 1(c) are for two cases: using $r_{\text{av}}$ (corresponding to $S{F_{r}}^{\Delta }$ ) or setting $r_{\text{av}}=r$ (corresponding to $S{F_{r}}^{\delta }$ ) in Eqs. (35)–(37). $\text{MTS}{F_{r}}^{\Delta }$ and $VS{F_{r}}^{\Delta }$ in IM have no dependence on *r*, and there is a negligible dependence in SM. Therefore, the errors resulting from using *r* instead of $r_{\text{av}}$ are zero in IM and negligible in SM.

### 3.2. Effect of errors in baseline values of $\mu_{a},\mu_{s}^{\prime }$ , or *DPF*

The presented equations for parameter recovery require knowledge of the baseline $\mu_{a},\mu_{s}^{\prime }$ , and *DPF*. Here, we examine how errors in these parameters affect the recovery of $\Delta \mu_{a}$ .

Figure 2 shows the errors in the recovered $\Delta \mu_{a}$ due to errors in the baseline $\mu_{a}$ or $\mu_{s}^{\prime }$ , for different magnitudes of $\Delta \mu_{a}$ (10, 20, and 30%, corresponding to 0.01, 0.02, and 0.03 cm^−1^, respectively). Consistent with the results in Fig. 1, the errors in recovered $\Delta \mu_{a}$ depend on the magnitude of $\Delta \mu_{a}$ when using $S{F_{a}}^{\delta }$ , but not when using $S{F_{a}}^{\Delta }$ .

When $\mu_{a}$ is overestimated by 100% (Fig. 2(a)), the $\Delta \mu_{a}$ values recovered using $MP{P_{a}}^{\Delta }$ are overestimated by about 30%, corresponding to recovered values of approximately 0.013, 0.026, and 0.039 cm^−1^ for the three magnitudes of $\Delta \mu_{a}$ . When $\mu_{s}^{\prime }$ is overestimated by 100% (Fig. 2(d)), the $\Delta \mu_{a}$ values recovered using $MP{P_{a}}^{\Delta }$ are underestimated by about 30%. If the percentage errors in $\mu_{a}$ and $\mu_{s}^{\prime }$ are similar, the error in the recovered $\Delta \mu_{a}$ will be close to zero, as expected, since $MP{P_{a}}^{\Delta }$ (Eq. (17)) is nearly proportional to the ratio $\sqrt{\mu_{s}^{\prime }\;/\;\mu_{a}}$ . When using $MP{P_{a}}^{\Delta }$ , the errors in the recovered $\Delta \mu_{a}$ remain nearly independent of the magnitude of $\Delta \mu_{a}$ despite large errors in the baseline $\mu_{a}$ and $\mu_{s}^{\prime }$ , unlike when using $MP{P_{a}}^{\delta }$ .

The derived equations for the MBLL (Eqs. (44) and (46)) contain the baseline $\mu_{a}$ and $\mu_{s}^{\prime }$ , which cannot be estimated using only continuous-wave measurement [6]. However, large errors in these parameters have a negligible effect on the recovered $\Delta \mu_{a}$ when the value of *DPF* is known accurately (Fig. 2(a, d)).

When using $\text{MTS}F_{a}$ (Fig. 2(b, e)) or $VSF_{a}$ (Fig. 2(c, f)), errors in the baseline $\mu_{s}^{\prime }$ and especially $\mu_{a}$ can lead to large errors in the recovered $\Delta \mu_{a}$ . For example, $\Delta \mu_{a}$ recovered using $VS{F_{a}}^{\Delta }$ is overestimated by approximately 60% when $\mu_{a}$ is overestimated by 20%. Therefore, when using $\text{MTS}F_{a}$ or $VSF_{a}$ , the baseline optical properties should be known accurately for reliable recovery of $\Delta \mu_{a}$ .

Figure 3 shows the errors in the recovered $\Delta \mu_{a}$ due to errors in the assumed value of *DPF* when using the MBLL (Eq. (41)), or the presented Eqs. (44) and (46) for IM and SM, respectively. Figure 3(a) presents results for a range of errors in the *DPF* value, and Fig. 3(b) presents results for a range of $\Delta \mu_{a}$ magnitudes.

A percentage error in *DPF* leads to a similar but negative percentage error in the recovered $\Delta \mu_{a}$ (Fig. 3(a)), as expected since *DPF* is a scaling factor. When *DPF* is overestimated by 20%, the recovered $\Delta \mu_{a}$ values are underestimated by approximately 18%. When using the MBLL, the percentage error in the recovered $\Delta \mu_{a}$ depends on the magnitude of $\Delta \mu_{a}$ , in contrast to using the derived Eqs. (44) and (46).

Notably, the values of $\Delta \mu_{a}$ recovered using Eq. (45) are similar to those recovered using Eq. (46). For a 100%, 200%, and 300% $\Delta \mu_{a}$ in SM, Eq. (45) recovers $\Delta \mu_{a}$ as 104%, 212%, and 322%, respectively. Figure 3(b) shows the corresponding results up to 100%. For comparison, the corresponding values of $\Delta \mu_{a}$ recovered using the MBLL (Eq. (41)) are 85%, 153%, and 211%, respectively. Therefore, Eq. (45), which is derived for IM, may also be used for SM measurements with small errors, especially when *DPF* is known accurately (Fig. 3(b)).

### 3.3. Spuriously recovered $\Delta \mu_{a}$ due to $\Delta \mu_{s}^{\prime }$ (or $\Delta \mu_{s}^{\prime }$ due to $\Delta \mu_{a}$ )

**Fig. 4.**
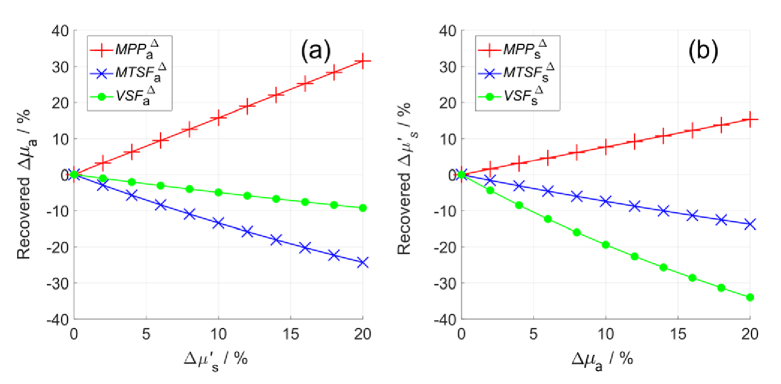
Spuriously recovered $\Delta \mu_{a}$ due to $\Delta \mu_{s}^{\prime }$ (a), and $\Delta \mu_{s}^{\prime }$ due to $\Delta \mu_{a}$ (b). Recovery in (a) uses the $S{F_{a}}^{\Delta }$ (Eqs. (20)–(22)), and recovery in (b) uses the $S{F_{s}}^{\Delta }$ equations (Eqs. (32)–(34)). The ground-truth values are $\Delta \mu_{a}=0$ in (a) and $\Delta \mu_{s}^{\prime }=0$ in (b).

The sensitivity factors for recovery of $\Delta \mu_{a}$ or $\Delta \mu_{s}^{\prime }$ assume that only one optical property changes, since a single measurand cannot be used to recover changes in multiple parameters. Figure 4 shows the spuriously recovered $\Delta \mu_{a}$ or $\Delta \mu_{s}^{\prime }$ arising from unaccounted changes in the other optical property.

A 20% increase in $\mu_{s}^{\prime }$ leads to a spuriously recovered $\Delta \mu_{a}$ of approximately 30%, –25%, and –10% when recovered from $\Delta A,\Delta m_{1}$ , and ΔV , respectively (Fig. 4(a)). Similarly, a 20% increase in $\mu_{a}$ leads to a spuriously recovered $\Delta \mu_{s}^{\prime }$ of approximately 15%, –15%, and −35% when recovered from $\Delta A,\Delta m_{1}$ , and ΔV , respectively (Fig. 4(b)). These results were obtained for *r* = 3 cm. For *r* = 1.5 cm, the magnitudes of the spuriously recovered changes were approximately 25% lower than those obtained for *r* = 3 cm (results not shown). When both optical properties change, they can be recovered from changes in two or more measurands using Eq. (47) in the Discussion.

## 4. Discussion

In the present study, we extended previously established sensitivity factor equations for infinitesimal changes in $\mu_{a}(S{F_{a}}^{\delta })$ [25] to finite changes, yielding the $S{F_{a}}^{\Delta }$ equations. These equations can be used to recover $\Delta \mu_{a}$ in homogeneous media from changes in statistical moments of DTOFs, which are typically acquired with time-resolved NIRS. By correcting for finite changes, $SF^{\Delta }$ improves recovery accuracy for $\Delta \mu_{a}$ and enables accurate recovery of large $\Delta \mu_{a}$ , as shown in Fig. 1. In addition, we derived sensitivity factor equations for changes in $\mu_{s}^{\prime }$ and *r*. Recovery using $SF^{\delta }$ underestimates positive changes, as shown in Fig. 1, and overestimates negative changes (not shown). This forms the basis of the finite-change correction used to derive $SF^{\Delta }$ . The $SF^{\Delta }$ equations reduce to the $SF^{\delta }$ equations in the limit of infinitesimal changes.

The modified Beer-Lambert law (MBLL) is valid for infinitesimal changes in $\mu_{a}$ due to the assumption that *DPF* remains constant with respect to $\Delta \mu_{a}$ , which is not valid according to the equation for *DPF* (Eq. (42)). We present equations for the MBLL that are valid for finite changes (Eq. (44) for IM and Eq. (46) for SM), which can be applied in data analysis algorithms used for continuous-wave NIRS measurements. They require knowledge of the baseline values of $\mu_{a}$ and $\mu_{s}^{\prime }$ , in addition to *DPF*, and these parameters cannot be estimated from continuous-wave measurements with a single source-detector pair [6]. However, errors in the baseline $\mu_{a}$ or $\mu_{s}^{\prime }$ (up to 100%) have a negligible effect (a few percent) on the recovered $\Delta \mu_{a}$ (Fig. 2(a, d)). Therefore, baseline optical properties can be assumed within the range of values in literature for the measured tissue, e.g., *μ*_a_ = 0.1 cm^−1^ and $\mu_{s}^{\prime }=10\;{\text{cm}}^{-1}$ for many biological tissues [13–15], resulting in only small percentage errors in the recovered $\Delta \mu_{a}$ . The value of *DPF* is typically taken from literature [38], and a percentage error in *DPF* leads to a similar percentage error in the recovered $\Delta \mu_{a}$ (Fig. 3), as expected since *DPF* is a scaling factor.

The parameter recovery based on $SF^{\Delta }$ can be done using nonlinear solvers (e.g., *fsolve* in MATLAB). Alternatively, for some of the $S{F_{a}}^{\Delta }$ equations we found analytical solutions for $\Delta \mu_{a}$ , which are presented in the Appendix and do not require nonlinear solvers. The $SF^{\Delta }$ equations require knowledge of the baseline optical properties, and large errors in these parameters can lead to large errors in the recovered $\Delta \mu_{a}$ (Fig. 2). However, if the IRF is measured, the baseline $\mu_{a}$ and $\mu_{s}^{\prime }$ can be estimated using the moments approach [24] or by fitting a solution of the DE to the baseline DTOF [27] . Although this approach allows estimating $\mu_{a}$ and $\mu_{s}^{\prime }$ at each time point, analysis of changes in moments is computationally simpler and advantageous for monitoring dynamic changes. The baseline DTOF can be acquired over a longer time to estimate baseline $\mu_{a}$ and $\mu_{s}^{\prime }$ with higher precision, after which changes in moments can be used to recover subsequent dynamic changes in $\mu_{a}$ .

The $SF^{\Delta }$ introduce a finite-change correction to the established data analysis approach based on $SF^{\delta }$ . Therefore, we expect their practical robustness to be similar. However, a separate stuty is needed to assess the effect of experimental factors including photon-count noise, DTOF cut-off region, instrument response function (IRF), and mismatch between the DE model and the measured medium. Theoretical uncertainty in DTOF moments due to photon noise, and the resulting uncertainty in recovered $\Delta \mu_{a}$ , has previously been analyzed for optimizing source-detector distance *r* [40]. A recent study showed the improved performance of time-domain moment techniques in comparison to conventional continuous-wave approaches in recovering hemodynamic responses [28]. A quantitative performance comparison using measured data showed that DTOF moments outperform measurands based on photon counts in time windows and their ratios [34].

All derived equations assume that the DTOF extends to infinite arrival time of photons. The calculated moments depend on the chosen cut-off noise region on the time axis, which is typically set at 1% of the maximum of the DTOF. A correction procedure was proposed to correct the values of moments due to a limited cut-off region [41]. In our previous study [42], in data analysis we compared changes in moments of the measured DTOFs to changes in moments of the analytically obtained DTOFs convolved with the IRF, such that both measured and analytically obtained moments are calculated using the same cut-off regions. DTOFs for homogeneous and multi-layered media can be obtained using a publicly available MATLAB script [42], which implements the solution of the DE presented by Liemert et al. [43].

This study considers a homogeneous medium, whereas measurements on layered tissue structures, such as the head, should use a multi-layered model [44,45]. Perturbation-based sensitivity factors $SF^{\delta }$ can be obtained for any geometry and complex medium using Monte Carlo simulations, e.g., for MRI-based head models [46]. Equation (10) provides a general way to recover finite changes using $SF^{\delta }$ calculated for both the baseline and perturbed states. When analytical expressions for $SF^{\delta }$ are available, they can be substituted into Eq. (10) to derive $SF^{\Delta }$ equations, as done in this study. When only numerical $SF^{\delta }$ values are available, such as from simulations, a possible strategy is to use Eq. (10) iteratively: starting with $\Delta \mu_{a}=0$ , estimate $\Delta \mu_{a}$ , recalculate $SF^{\delta }$ for the perturbed state, and repeat until the estimated $\Delta \mu_{a}$ changes negligibly between iterations. The convergence and accuracy of such an approach would need to be verified for the specific geometry, medium, and measurand.

In measurements on tissues, $\mu_{s}^{\prime }$ is typically assumed constant because separating $\Delta \mu_{a}$ from $\Delta \mu_{s}^{\prime }$ is challenging [47]. However, $\Delta \mu_{s}^{\prime }$ may be caused by changes in total hemoglobin concentration, which changes the number of scatterers within the volume of tissue interrogated by photons [48]. Studies carried out on rat brain slices have shown that $\Delta \mu_{s}^{\prime }$ can appear in line with $\Delta \mu_{a}$ [49–51]. In a number of studies, $\Delta \mu_{s}^{\prime }$ following fast neuronal activity have been reported in animals [52] and humans [53,54]. Changes in two or more measurands *M*, i.e., $\Delta A,\Delta m_{1}$ , and/or ΔV , allow recovering both $\Delta \mu_{a}$ and $\Delta \mu_{s}^{\prime }$ . Note that $SF^{\Delta }$ equations were derived for changes in only one optical property while the other is assumed constant. The effects of changes in the other optical property are shown in Fig. 4. However, both $\Delta \mu_{a}$ and $\Delta \mu_{s}^{\prime }$ can be simultaneously recovered by using each $SF^{\Delta }$ for a change in only one parameter.

(47) $$\Delta M=S{F_{a}}^{\Delta }(\mu_{a},\mu_{s}^{\prime })\cdot \Delta \mu_{a}+S{F_{s}}^{\Delta }\left(\mu_{a}+\Delta \mu_{a},\mu_{s}^{\prime }\right)\cdot \Delta \mu_{s}^{\prime }$$

Time-resolved measurements allow estimating the pathlength traveled by detected photons from their arrival time. When the MBLL is applied to photon counts with the same arrival time, it remains valid for any magnitude of $\Delta \mu_{a}$ . The mean pathlength within a time channel is known as the time-dependent *MPP* (*TMPP*) [55]. This approach is also referred to as the microscopic BLL [17], which corresponds to applying the BLL to pathlength-resolved light intensity. This enables recovery of $\Delta \mu_{a}$ at multiple depths, since late photons, on average, probe deeper regions of the medium than early photons [55]. Similarly, sensitivity factors for higher-order moments have increased sensitivity to deeper compartments, allowing moments-based methods to recover $\Delta \mu_{a}$ in multiple layers [25,42,56]. The influence of changes in superficial layers is an inherent major challenge in brain functional studies [44].

The derived equations rely on the diffusion equations (DE) for IM and SM, which approximate the radiative transfer equation (RTE), which is a specific case of the more general transport theory applied to light propagation [1,57]. We did not introduce new assumptions and the derived $SF^{\Delta }$ equations remain valid for any magnitude changes, as long as the DE remains valid (Eqs. (1) and (2)). The DE relies on the diffusion approximation, which assumes that $\mu_{s}^{\prime }$ is much greater than $\mu_{a}$ , and either the photon arrival time (for time-resolved measurements) or the source-detector distance *r* (for non-time-resolved measurements) is sufficiently large for light propagation to be diffusive [1]. In measurements on tissues, $\mu_{s}^{\prime }$ is typically two orders of magnitude higher than $\mu_{a}$ in the NIR range [13–15], hence the DE remains valid for large $\Delta \mu_{a}$ , provided that scattering continues to dominate absorption. The presented finite-change correction may also be extended to other geometries, such as slab (transmission geometry), or for multi-layer tissue models. Such extensions would need to be derived and validated separately.

## 5. Conclusions

We derived equations for sensitivity factors that accurately relate ΔA and changes in the statistical moments of DTOFs ( $\Delta m_{1}$ and ΔV ) to finite (even large) $\Delta \mu_{a},\Delta \mu_{s}^{\prime }$ , and Δr in infinite medium IM and semi-infinite medium SM geometries. We verified the equations and showed the improvement in the accuracy of parameter recovery compared to the established perturbation-based approaches (Fig. 1).

We derived equations for the modified Beer–Lambert law (MBLL) that are valid for finite $\Delta \mu_{a}$ in IM and SM (Eqs. (44) and (46)). These equations require knowledge of the baseline $\mu_{a}$ and $\mu_{s}^{\prime }$ , in addition to the differential pathlength factor *DPF*. However, even a 100% relative error in $\mu_{a}$ results in only a few percent error in the recovered $\Delta \mu_{a}$ , whereas errors in $\mu_{s}^{\prime }$ have an even smaller effect (Fig. 2). Therefore, typical values of $\mu_{a}$ and $\mu_{s}^{\prime }$ from literature can be assumed for tissue measurements.

The sensitivity factor equations require knowledge of the baseline $\mu_{a}$ and $\mu_{s}^{\prime }$ , and large errors in these parameters can lead to large errors in the recovered parameter changes (Fig. 2). Therefore, optical properties at baseline need to be estimated, after which subsequent dynamic changes can be recovered using sensitivity factors. Changes in the other optical property lead to spurious changes in the recovered optical property (Fig. 4), in which case both $\Delta \mu_{a}$ and $\Delta \mu_{s}^{\prime }$ should be recovered from changes in two or more measurands (Eq. (47)).

Future experimental work should assess the robustness of the presented equations to photon noise, instrumental effects, DTOF cut-off region, and model mismatch. However, the robustness is expected to be comparable to that of the established approach based on sensitivity factors for infinitesimal changes, since the presented equations only introduce a finite-change correction.

The presented equations can improve estimation of changes in optical properties in NIRS. The derivation approach may also be useful for deriving finite-change corrections to other perturbation-based equations in diffuse optics, although they would need to be derived and validated separately.

## Data Availability

Data shown in this article are generated using analytical formulas as presented. Therefore, no data is available.

## Appendix

The sensitivity factor equations for finite absorption changes $S{F_{a}}^{\Delta }$ (Eqs. (17)–(22)) were derived by substituting the below expressions for $SF_{a}(\mu_{a})$ and $SF_{a}({\mu_{a}}^{*})$ into Eq. (10). The two expressions used to derive $MP{P_{a}}^{\Delta }$ are the $MP{P_{a}}^{\delta }$ equation (Eq. (11)) for the baseline $\mu_{a}$ and the perturbed state ${\mu_{a}}^{*}$ , where ${\mu_{a}}^{*}=\mu_{a}+\Delta \mu_{a}$ , and $\mu_{e}=\sqrt{3\mu_{a}\mu_{s}^{\prime }}$ , and ${\mu_{e}}^{*}=\sqrt{3{\mu_{a}}^{*}\mu_{s}^{\prime }}$ . The same approach was used to derive all $S{F_{s}}^{\Delta }$ equations for $\Delta \mu_{s}^{\prime }$ . To derive $\text{MTS}{F_{a}}^{\Delta }$ and $VS{F_{a}}^{\Delta }$ for IM, both expressions $SF_{a}(\mu_{a})$ and $SF_{a}({\mu_{a}}^{*})$ contain both $\mu_{a}$ and ${\mu_{a}}^{*}$ .

To derive $MP{P_{a}}^{\Delta }$ for IM (Eq. (17)), the expressions used are:

(48)
$$SF_{a}(\mu_{a})=\frac{r\sqrt{3\mu_{s}^{\prime }}}{2}\left(\frac{1}{\sqrt{\mu_{a}}}\right)\;\;SF_{a}({\mu_{a}}^{*})=\frac{r\sqrt{3\mu_{s}^{\prime }}}{2}\left(\frac{1}{\sqrt{{\mu_{a}}^{*}}}\right)$$

To derive $MP{P_{a}}^{\Delta }$ for SM (Eq. (20)), the expressions used are:

(49)
$$SF_{a}(\mu_{a})=\frac{r\sqrt{3\mu_{s}^{\prime }}}{2}\left(\frac{1}{\sqrt{\mu_{a}}}\right)\left(\frac{r}{r\mu_{e}+1}\right)\;SF_{a}({\mu_{a}}^{*})=\frac{r\sqrt{3\mu_{s}^{\prime }}}{2}\left(\frac{1}{\sqrt{{\mu_{a}}^{*}}}\right)\left(\frac{r}{r{\mu_{e}}^{*}+1}\right)$$

To derive $\text{MTS}{F_{a}}^{\Delta }$ for IM (Eq. (18)), the expressions used are:

(50)
$$SF_{a}(\mu_{a})=-\frac{1}{4c}\frac{r\sqrt{3\mu_{s}^{\prime }}}{\sqrt{\mu_{a}{\mu_{a}}^{*}}}\left(\frac{1}{\sqrt{\mu_{a}}}\right)\;SF_{a}({\mu_{a}}^{*})=-\frac{1}{4c}\frac{r\sqrt{3\mu_{s}^{\prime }}}{\sqrt{\mu_{a}{\mu_{a}}^{*}}}\left(\frac{1}{\sqrt{{\mu_{a}}^{*}}}\right)$$

To derive $VS{F_{a}}^{\Delta }$ for IM (Eq. (19)), the expressions used are:

(51)
$$SF_{a}(\mu_{a})=-\frac{3}{8c^{2}}\frac{r\sqrt{3\mu_{s}^{\prime }}}{\mu_{a}{\mu_{a}}^{*}}\left(\frac{1}{\sqrt{\mu_{a}}}\right)\;SF_{a}({\mu_{a}}^{*})=-\frac{3}{8c^{2}}\frac{r\sqrt{3\mu_{s}^{\prime }}}{\mu_{a}{\mu_{a}}^{*}}\left(\frac{1}{\sqrt{{\mu_{a}}^{*}}}\right)$$

To derive $\text{MTS}{F_{a}}^{\Delta }$ and $VS{F_{a}}^{\Delta }$ for SM, their equations for IM are used together with the ratios that distinguish the moment equations (Eqs. (4)–(6)) for IM from those for SM. To derive $\text{MTS}{F_{a}}^{\Delta }$ for SM (Eq. (21)), the expressions used are:

(52)
$$SF_{a}(\mu_{a})=\text{MTS}{F_{a,\text{IM, Eq.18}}}^{\Delta }{\left(\frac{r\mu_{e}}{r\mu_{e}+1}\right)}^{2}\;SF_{a}({\mu_{a}}^{*})=\text{MTS}{F_{a,\text{IM, Eq.18}}}^{\Delta }{\left(\frac{r{\mu_{e}}^{*}}{r{\mu_{e}}^{*}+1}\right)}^{2}$$

To derive $VS{F_{a}}^{\Delta }$ for SM (Eq. (22)), the $SF_{a}(\mu_{a})$ and $SF_{a}({\mu_{a}}^{*})$ expressions used are:

(53)
$$VS{F_{a,\text{IM, Eq. 19}}}^{\Delta }\left(\frac{{\mu_{e}}^{2}r^{2}(r\mu_{e}+1/3)}{{\left(r\mu_{e}+1\right)}^{3}}\right)\;\text{and}\;VS{F_{a,\text{IM, Eq. 19}}}^{\Delta }\left(\frac{{\mu_{e}}^{*2}r^{2}(r{\mu_{e}}^{*}+1/3)}{{\left(r{\mu_{e}}^{*}+1\right)}^{3}}\right)$$

Nonlinear solvers, such as *fsolve* function in MATLAB, should be used to recover $\Delta \mu_{a}$ from the measured changes in moments using the corresponding $S{F_{a}}^{\Delta }$ equations (Eqs. (17)–(19)). Alternatively, for some of these equations we found analytical solutions for $\Delta \mu_{a}$ , which do not require nonlinear solvers.

When using $MP{P_{a}}^{\Delta }$ for IM (Eq. (17)), the closed-form solution for $\Delta \mu_{a,\text{IM}}$ is:

(54)
$$\Delta \mu_{a,\text{IM}}=\frac{2\sqrt{\mu_{a}}\Delta A}{r\sqrt{3\mu_{s}^{\prime }}}+\frac{\Delta A^{2}}{3\mu_{s}^{\prime }r^{2}}$$

The first term is equivalent to using $MP{P_{a}}^{\delta }$ (Eq. (11)). The second term, which contains $\Delta A^{2}$ , is an order of magnitude smaller than the first term for a 10% $\Delta \mu_{a}$ and typical tissue optical properties $\mu_{a}=0.1\;{\text{cm}}^{-1}$ and $\mu_{s}^{\prime }=10\;{\text{cm}}^{-1}$ . Equation (54) is the same as Eq. (45), but with *DPF* expressed in terms of optical properties.

The $MP{P_{a}}^{\Delta }$ equation for SM (Eq. (20)) is too complex to find an exact closed-form solution for $\Delta \mu_{a,\text{SM}}$ . However, we found an approximate solution from numerical inversion, which is practically as accurate as the exact solution:

(55)
$$\begin{array}{ll} \Delta \mu_{a,\text{SM}} & =\frac{\Delta A}{6r^{2}\mu_{s}^{\prime 2}}[2r\sqrt{3\mu_{a}}\mu_{s}^{\prime 3/2}+\mu_{s}^{\prime }\Delta A+4\mu_{s}^{\prime }\\ & \;+{\left(4r\sqrt{3\mu_{a}}\mu_{s}^{\prime 5/2}\Delta A+12r^{2}\mu_{a}\mu_{s}^{\prime 3}+\mu_{s}^{\prime 2}\Delta A^{2}+8\mu_{s}^{\prime 2}\Delta A\right)}^{1/2}] \end{array}$$

When using $\text{MTS}{F_{a}}^{\Delta }$ for IM (Eq. (18)), the closed-form solution for $\Delta \mu_{a,\text{IM}}$ is:

(56)
$$\Delta \mu_{a,\text{IM}}=\frac{{\mu_{a}}^{2}}{{\left(\frac{r\sqrt{3\mu_{s}^{\prime }}}{2c\Delta m_{1}}+\sqrt{\mu_{a}}\right)}^{2}}-\frac{2{\mu_{a}}^{3/2}}{\frac{r\sqrt{3\mu_{s}^{\prime }}}{2c\Delta m_{1}}+\sqrt{\mu_{a}}}$$

When using $VS{F_{a}}^{\Delta }$ for IM (Eq. (19)), we found an approximate solution from numerical inversion, which is practically as accurate as the exact solution:

(57)
$$\Delta \mu_{a,\text{IM}}=\frac{3r\sqrt{3\mu_{s}^{\prime }}}{4c^{2}\sqrt{\mu_{a}}\Delta V}-\mu_{a}+\frac{27r^{2}\mu_{s}^{\prime }-9r^{3/2}\mu_{s}^{\prime 3/4}{\left(16\sqrt{3}c^{2}{\mu_{a}}^{3/2}\Delta V+9r\sqrt{\mu_{s}^{\prime }}\right)}^{1/2}}{32c^{4}{\mu_{a}}^{2}\Delta V^{2}}$$

Equation (43) shows the relation between $MP{P_{a}}^{\Delta }$ and $MP{P_{a}}^{\delta }$ in IM, which was used to derive the finite-change equations for the MBLL. For completeness, we present the relation between $\text{MTS}{F_{a}}^{\Delta }$ and $\text{MTS}{F_{a}}^{\delta }$ (Eq. (58)), and between $VS{F_{a}}^{\Delta }$ and $VS{F_{a}}^{\delta }$ (Eq. (59)) in the IM. Equations (43), (58), and (59) have a common term (in the first brackets), and the equations for higher-order moments contain an additional term (in the second brackets). This additional term reflects the greater deviation from linearity of higher-order moment changes with respect to changes in $\mu_{a}$ .

(58)
$$\text{MTS}{F_{a,\text{IM}}}^{\Delta }=\text{MTS}{F_{a,\text{IM}}}^{\delta }\left(\frac{2}{1+\sqrt{1+\Delta \mu_{a}/\mu_{a}}}\right){\left(\frac{1}{1+\Delta \mu_{a}/\mu_{a}}\right)}^{1/2}$$

(59)
$$VS{F_{a,\text{IM}}}^{\Delta }=VS{F_{a,\text{IM}}}^{\delta }\left(\frac{2}{1+\sqrt{1+\Delta \mu_{a}/\mu_{a}}}\right)\left(\frac{1}{1+\Delta \mu_{a}/\mu_{a}}\right)$$
